# Pleural Fluid Cholesterol in Differentiating Exudative and Transudative Pleural Effusion

**DOI:** 10.1155/2013/135036

**Published:** 2013-01-10

**Authors:** A. B. Hamal, K. N. Yogi, N. Bam, S. K. Das, R. Karn

**Affiliations:** Department of Internal Medicine, Tribhuvan University Teaching Hospital (TUTH), Maharajgunj, Kathmandu, Nepal

## Abstract

*Objectives*. To study the diagnostic value of pleural fluid cholesterol in differentiating transudative and exudative pleural effusion. To compare pleural fluid cholesterol level for exudates with Light's criteria. *Design*. Cross sectional descriptive study. *Settings*. Medical wards of Tribhuvan University Teaching Hospital. *Methods*. Sixty two cases of pleural effusion with definite clinical diagnosis admitted in TUTH were taken and classified as transudates (19) and exudates (43). The parameters pleural fluid protein/serum protein ratio (pfP/sP), pleural fluid LDH/ serum LDH ratio, pleural fluid LDH (pfLDH) and pleural fluid cholesterol (pCHOL) were compared with clinical diagnosis with regard to their usefulness for distinguishing between pleural exudates and transudates. *Results*. The pCHOL values determined were 1.92 ± 0.75 for exudates, 0.53 ± 0.28 for transudates, the differences between the transudates and others are statistically significant (*P* < 0.0001). It is seen that pfP/sP ratio has a sensitivity of 81.4% and specificity of 82.6%; pfLDH/sLDH ratio has a sensitivity of 86% and specificity of 94.7% and pCHOL with sensitivity of 97.7% and specificity of 100% for differentiating exudative and transudative PE. *Conclusion*. The determination of pCHOL is of great value for distinguishing between pleural exudates and transudates and should be included in routine laboratory analysis of pleural effusion.

## 1. Introduction 

Light et al. in 1972 found criteria to have sensitivity and specificity of 99% and 98%, respectively, for differentiating transudative and exudative PEs (ratio of protein in pleural fluid and serum >0.5; ratio of LDH in pleural fluid and serum >0.6; pleural fluid LDH >2/3rd of upper limit of serum LDH) [1].

But the other investigators could only reproduce specificities of 70–86% using Light's criteria. Also it is found that 25% of patients with transudates pleural effusion are mistakenly identified as having exudative effusion by Light's criteria. In cases of heart failure on diuretic therapy, the transudative PE has high protein [2].

Pleural fluid cholesterol can be used to classify exudates and transudates as it misclassifies fewer cases than any other Light's parameters [3]. From meta-analysis, Heffner et al. 2002 have identified pleural effusion of exudative type with at least one of the following conditions [4].

- Pleural fluid protein >2.9 gm/dL.
- Pleural fluid cholesterol >45 mg/dL (1.16 mmol/L).
- Pleural fluid LDH >2/3rd of upper limit of serum.

Pleural cholesterol is thought to be derived from degenerating cells and vascular leakage from increased permeability. Though the cause of the rise in cholesterol levels in pleural exudates is unknown, two possible explanations have been put forward. 

According to the first, the cholesterol is synthesized by pleural cells themselves for their own needs [5] (extrahepatic synthesis of cholesterol is now known to be much greater than was once thought, depends on the metabolic needs of cells, and is in dynamic equilibrium with cholesterol supply by LDL and cholesterol removal by HDL) [6], and the concentration of cholesterol in pleural cavity is increased by the degeneration of leukocytes and erythrocytes, which contain large quantities.

The second possible explanation is that pleural cholesterol derives from plasma; some 70 percent of plasma cholesterol is bound to low density, high molecular weight lipoproteins (LDL), and the rest to HDL or very low density lipoproteins (VLDL), and the increased permeability of pleural capillaries in pleural exudate patients would allow plasma cholesterol to enter the pleural cavity.

The reason to select the cutoff value of pleural fluid cholesterol as 45 (1.16 mmol/L) is that this cutoff value eliminates the possibility of being equivocal to transudates and exudates, and measurement of pleural cholesterol >45 mg/dL (1.16 mmol/L) has been used to improve the accuracy of differentiating transudative and exudative effusions [7]. 

## 2. Methods

Sample size of 62 consecutive pleural effusion cases that fulfilled the inclusion criteria and were admitted in the Department of Internal Medicine, TUTH were taken. The study period was conducted for one year from July 2010 to August 2011.

### 2.1. Inclusion Criteria

1. Age ≥ 16 yrs,
2. patients giving consent,
3. patients with definite clinical diagnosis and pleural effusion evidenced by radiological imaging.

### 2.2. Exclusion Criteria

1. Patients not willing to participate in the study,
2. age < 16 years,
3. patients without definite clinical diagnosis,
4. patients with pulmonary embolism or renal insufficiency with pleural effusion,
5. patients previously diagnosed and already on treatment.

### 2.3. Study Procedure

After a detailed history and clinical examination, chest X-ray was done to localize pleural effusion. Diagnostic tapping of the pleural fluid was done in every case, and the help of ultrasonography of chest to localize the fluid was taken in some cases. All pleural fluid samples were tested for cell count, protein, glucose, LDH, pCHOL, Gram stain, bacterial culture, acid fast stain, and cytology. A concomitant blood sample was taken and tested for counts and biochemical parameters such as protein and LDH. Further investigations, such as computed tomography scan of chest, bronchoscopy, and fine needle aspiration cytology (FNAC), were also done to determine etiology of pleural effusion when needed. 

The first sample of pleural fluid obtained in each patient was considered for analysis. Protein was measured by the biuret method, LDH by UV spectrophotometry at 37°C and 340 nm [8], and cholesterol with the Boehringer-Mannheim enzymatic method CHOD PAP (cholesterol oxidase peroxidise) [9]. 

Clinical diagnosis (i.e., etiological diagnosis) was made, and the pleural fluid parameters were analyzed with it. The following evidences were used to include or exclude the cases [10].

1. Congestive heart failure: presence of clinical features (increased jugular venous pulse, tachycardia, and ventricular gallop) with cardiomegaly or echocardiac evidence of cardiac dysfunction.
2. Renal diseases: elevated urea (>20 mmol/L) or creatinine > 167 micomol/L with or without signs or symptoms of fluid overload.
3. Malignancy: confirmed by cytology or histological proof of malignant tumor and in absence of all other conditions associated with pleural effusion.
4. Liver cirrhosis: positive ultrasonography or CT findings with clinical, and lab evidence of hepatic derangements and portal hypertension.
5. Infective effusion: clear evidence of infection (positive microbiologic culture), elevated CRP or leukocytosis, or positive sputum stain.
6. Hypoalbuminemia: serum albumin < 20 gm/L.

Pleural effusions associated with congestive cardiac failure, hypoalbuminemia, and liver cirrhosis were classified as transudates and all others as exudates. Cases of renal diseases and pulmonary embolism were excluded.

Thus, pleural fluid was categorized as transudative and exudative pleural effusion on the basis of etiology which was contributed by clinical, imaging, and pathological evaluations. The pleural effusions were classified as exudative and transudative on the basis of etiological diagnosis, Light's criteria, and pCHOL (taken a cutoff value of 1.16 mmol/L or 45 mg/dL, given by Heffner et al. 2002) [4]. 

Quiroga et al. [11], using 45 mg/dL of cholesterol as the cutoff in 80 patients, also reported a sensitivity of 83% and a specificity of 100%. The statistical significance of the parameters for etiological diagnosis was measured to find their usefulness.

## 3. Observations and Results

**Figure 1:**
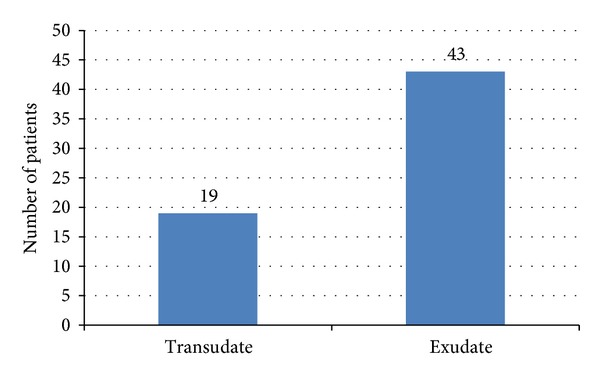
Distribution of type of pleural effusion.

A total of 62 patients with definite clinical diagnosis, eligible for the study, were included in which 30.6% (19) cases were transudates, and 69.4% (43) cases were exudates (Figure 1).

**Figure 2:**
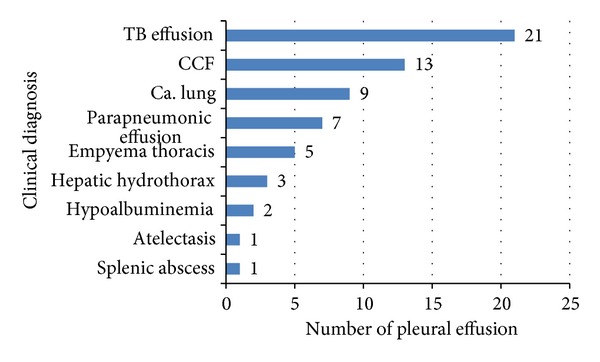
Distribution of causes (clinical diagnosis) of pleural effusion.

It is seen that tubercular effusion was the most common PE in the study. It counted 21 out of 62 cases (33.9%). Carcinoma lung was the second most common cause accounting for 14.5% (9), followed by parapneumonic effusion 11.3% (7), empyema thoracis 8.1% (5), hepatic hydrothorax 4.8% (3), hypoalbuminemia (2 cases), and 1 case each for atelectasis and splenic abscess. Transudates counted for 21% (13 cases) (see Figure 2).

**Figure 3:**
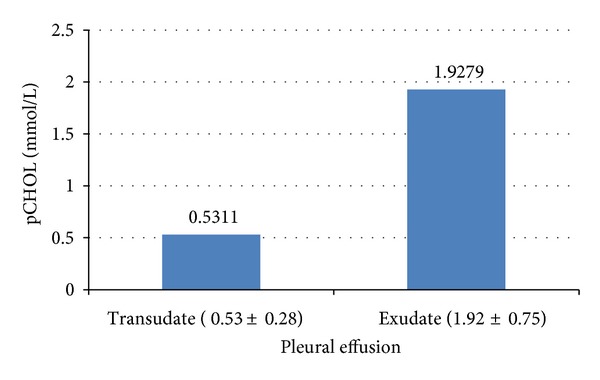
Mean values (±SD) of pCHOL (mmol/L) in type of pleural effusion.

**Figure 4:**
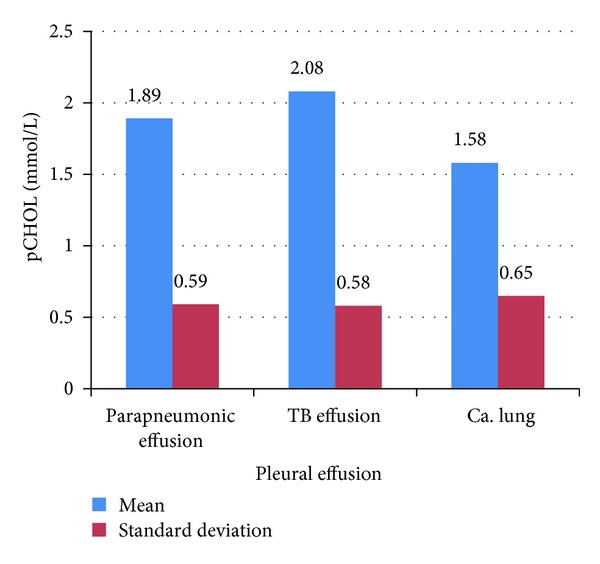
Mean values (±SD) of pCHOL (mmol/L) in different pleural effusion.

In this study, it is found that mean pCHOL level (mmol/L) was 1.92 ± 0.75 for exudates, 0.53 ± 0.28 for transudates, 1.81 ± 0.59 for parapneumonic effusion, 2.08 ± 0.58 for tubercular, and 1.58 ± 0.65 for malignancy as shown in Figures 3 and 4, respectively.

**Figure 5:**
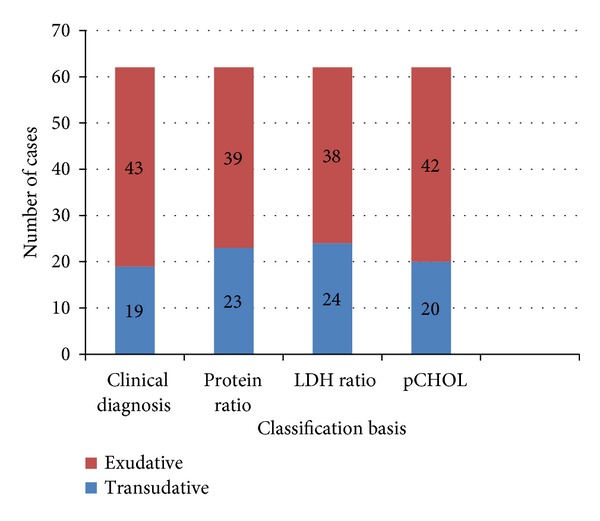
Cases classified by Light's criteria and pCHOL with clinical diagnosis.

It is seen that out of 62 cases (exudates 43 and transudates 19), protein ratio, as Light's parameter, identified 39 cases as exudates and 23 cases as transudates; LDH ratio identified 38 cases as exudates and 24 cases as transudates, while pCHOL identified 42 cases as exudate, and 20 cases as transudates (see Figure 5). 

It is seen that pfP/sP ratio has a sensitivity of 81.4% and specificity of 82.6%; pfLDH/sLDH ratio has a sensitivity of 86% and specificity of 94.7%, and pCHOL with sensitivity of 97.7% and specificity of 100% for differentiating exudative and transudative PEs. All these parameters have a significant *P* value that is, <0.0001 (see Table 1). 

Also on Pearson correlation test, pCHOL correlation is 0.963 and protein ratio (pfP/sP) is 0.591 which suggests that pCHOL is highly correlated than protein ratio with clinical diagnosis for exudate which is significant at the 0.01 level.

## 4. Discussion

In this study, a total of 62 patients, 19 with transudates and 43 with exudates, were considered according to the clinical diagnosis. The most frequent cause of pleural exudates is tuberculosis followed by lung cancer which is similar to the result of a study done in Malaysia where there is high incidence of tuberculosis [12]. Protein ratio identified exudates with a sensitivity of 81.4% and specificity of 82.6%. The pleural fluid to serum LDH ratio has a sensitivity and specificity of 86% and 94.7%, respectively. Also on Pearson correlation test, pCHOL correlation, and protein ratio (pfP/sP) are 0.963 and 0.591, respectively. It suggests that pCHOL is highly correlated than protein ratio with clinical diagnosis for exudate which is significant at the 0.01 level.

It is found that in transudates, parapneumonic, tubercular, and neoplastic pleural effusions, pCHOL levels were 0.53 ± 0.28 mmol/L, 1.81 ± 0.59 mmol/L, 2.08 ± 0.58 mmol/L, and 1.58 ± 0.65 mmol/L, respectively. With a classifying threshold of 1.16 mmol/L, pCHOL has a sensitivity of 97.7 percent and specificity of 100 percent for diagnosis of exudates with a PPV of 100 percent in this study. 

It was found that pCHOL criterion misclassified only one case of malignant effusion as transudate and that happened with the protein ratio of Light's criteria too. Similar findings have been reported by others, who suggested that the misclassified exudates had low cell component concentrations because the pleura had only recently been affected by the tumor [3, 13].

Other authors [14, 15] believe that a more likely explanation is that the pathogenesis of neoplastic exudates involves more than one mechanism more frequently than that of other kinds.

## 5. Conclusion

It is concluded that pCHOL has a better sensitivity, specificity, and PPV in differentiating transudates and exudates than the parameters of Light's criteria. This also avoids the plasma protein, sLDH and pleural fluid protein, and LDH. Therefore, it is, a more efficient, easier, and a more cost effective method to differentiate exudates from transudates. This study also suggests that determination of pCHOL should be in routine practice in cases of pleural effusion. 

## Figures and Tables

**Table 1:** Diagnostic comparison of PF parameters with clinical diagnosis.

Parameters	Sensitivity	Specificity	PPV	NPV	*P* value
Protein ratio	81.4%	82.6%	89.7%	70.4%	<0.0001
LDH ratio	86%	94.7%	97.4%	75%	<0.0001
pfLDH	100%	57.8%	84.3%	100%	<0.0001
pCHOL	97.7%	100%	100%	95%	<0.0001
